# Clinical features of persistent postural-perceptual dizziness with isolated otolith dysfunction as revealed by VEMP and vHIT findings

**DOI:** 10.3389/fneur.2023.1129569

**Published:** 2023-03-16

**Authors:** Masato Azami, Hiroaki Fushiki, Reiko Tsunoda, Tomohiko Kamo, Hirofumi Ogihara, Ryozo Tanaka, Takumi Kato

**Affiliations:** ^1^Otolaryngology, Mejiro University Ear Institute Clinic, Saitama, Japan; ^2^Department of Physical Therapy, Faculty of Health Sciences, Japan University of Health Sciences, Satte, Japan; ^3^Department of Physical Therapy, Faculty of Rehabilitation, Gunma Paz University, Takasaki, Japan; ^4^Division of Physical Therapy, Department of Rehabilitation, Faculty of Health Sciences, Nagano University of Health and Medicine, Nagano, Japan; ^5^Department of Physical Therapy, Faculty of Health Sciences, Mejiro University, Saitama, Japan

**Keywords:** persistent postural-perceptual dizziness, vestibular evoked myogenic potentials, isolated otolith dysfunction, Romberg ratio, dizziness handicap inventory

## Abstract

**Background:**

Persistent postural-perceptual dizziness (PPPD) is a relatively new disease entity, with diagnostic criteria published by the Bárány Society. PPPD is often preceded by a peripheral or central vestibular disorder. It is not clear how coexisting deficits due to preceding vestibular disorders affect PPPD symptoms.

**Objective:**

This study aimed to characterize the clinical features of PPPD with or without isolated otolith dysfunction using vestibular function tests.

**Methods:**

The study included 43 patients (12 males and 31 females) who were diagnosed with PPPD and completed oculomotor-vestibular function tests. The Dizziness Handicap Inventory (DHI), Hospital Anxiety and Depression Scale (HADS), Niigata PPPD Questionnaire (NPQ), and Romberg test for stabilometry were examined. The 43 patients with PPPD were classified into four categories based on vestibular evoked myogenic potential (VEMP) and video head impulse test (vHIT) results: normal function for both semicircular canals and otoliths (normal), isolated otolith dysfunction (iOtoDys), isolated semicircular canal dysfunction (iCanalDys), and dysfunction of both otoliths and semicircular canals (OtoCanalDys).

**Results:**

Among the 43 patients with PPPD, the iOtoDys group was the largest (44.2%), followed by the normal group (37.2%), iCanalDys group (9.3%), and OtoCanalDys group (9.3%). Eight of the 19 iOtoDys patients showed both abnormal cVEMP and oVEMP responses unilaterally or bilaterally (both sacculus and utriculus damage type), whereas 11 showed either an abnormal cVEMP or an abnormal oVEMP response (either sacculus or utriculus damage type). In a three-group comparison of the both sacculus and utriculus damage type, the either sacculus or utriculus damage type, and the normal group, the mean total, functional, and emotional DHI scores were significantly higher for the both sacculus and utriculus damage type than for the either sacculus or utriculus damage type. The Romberg ratio, a measure of stabilometry, was significantly higher for the normal group than for the both sacculus and utriculus damage type and the sacculus or utriculus damage type in the iOtoDys group.

**Conclusions:**

The coexistence of sacculus and utriculus damage may exacerbate dizziness symptoms in patients with PPPD. Determining the presence and extent of otolith damage in PPPD may provide useful information on the pathophysiology and treatment strategies of PPPD.

## 1. Introduction

Persistent postural-perceptual dizziness (PPPD) is a relatively new disease entity with diagnostic criteria published by the Bárány Society (1) and was introduced to the International Classification of Diseases in its 11th revision (ICD-11). PPPD is a common chronic dizziness-related disorder with a reported prevalence of up to 20% in patients with chronic dizziness (2). The main symptoms of PPPD are chronic non-spinning vertigo, dizziness, or unsteadiness. The symptoms are exacerbated by upright posture, active or passive movement, and complex visual stimuli, which cause significant distress or functional impairment (3).

PPPD is often preceded by a peripheral or central vestibular disorder. Benign paroxysmal positional vertigo (BPPV), vestibular migraine, and vestibular neuritis have been reported as background disorders in PPPD (2, 4, 5). Among prolonged deficits due to preceding vestibular disorders, isolated otolith dysfunction could be a potent factor in the development of PPPD, as it has been reported to be present in 20–46% of PPPD patients (4, 6, 7). It is not clear how the coexistence of isolated otolith dysfunction affects PPPD symptoms. This study aimed to characterize the clinical features of PPPD with or without isolated otolith dysfunction using vestibular function tests.

## 2. Materials and methods

### 2.1. Patients

Of the 63 patients diagnosed with PPPD at our university clinic between March 1, 2020, and February 28, 2022, 43 patients (12 males and 31 females) met the following inclusion criteria: (a) age between 20 and 80 years, (b) both vestibular evoked myogenic potentials (VEMPs) and the video head impulse test (vHIT) had been performed, and (c) no middle ear lesions. The diagnostic criteria for PPPD followed those outlined by the Bárány Society (1).

### 2.2. VEMP testing

Two types of cervical VEMP (cVEMP) and ocular VEMP (oVEMP) were measured to assess the saccular and utricular functions, respectively, using the Neuropack^®^ S3 Neurodiagnostic System (Nihon Kohden, Japan). For cVEMP recording, surface electrodes were placed on the upper half of the sternocleidomastoid muscle (active electrode), upper sternum (reference electrode), and forehead (ground electrode). Short tone bursts (500 Hz air-conducted, 105 dB SPL, rise/fall time = 1 ms, and plateau time = 2 ms) were delivered from a headphone. For oVEMP recording, the surface electrodes were placed on the skin 1 cm below (active electrode) and 3 cm below (reference electrode) the center of each lower eyelid, and on the forehead (ground electrode). Short tone bursts (500 Hz air-conducted, 105 dB SPL, rise/fall time = 1 ms, and plateau time = 2 ms) were delivered from the headphone.

The elicitation of cVEMP was confirmed when the characteristic p13-n23 (positive-negative waveform) appeared. The absence of cVEMP was determined when typical waveforms could not be elicited or were unrepeatable. The elicitation of oVEMP was confirmed when the characteristic n1-p1 (positive-negative waveform) appeared. The absence of oVEMP was established when typical waveforms could not be elicited or were unrepeatable. Normal ranges of latencies for p13 and n1 were considered 13.8–14.9 ms and 8.5–12.2 ms, respectively (8, 9). When a latency was outside its normal range, it was considered abnormal.

One measure of VEMP was the amplitude asymmetry ratio (AAR). AAR was calculated using the formula (AL – AR) / (AL + AR) × 100%, where AR is the normalized amplitude (p13-n23 or n1-p1) on the right side and AL is the normalized amplitude (p13-n23 or n1-p1) on the left side. In the present study, the upper limit standard of a normal AAR value of cVEMP was 34.0% (8) and that of oVEMP was 31.6% (9).

### 2.3. Video head impulse test (vHIT)

For the vHIT, all three semicircular canals were evaluated (Eye-See-Cam System Interacoustic, Denmark). The patient was asked to sit upright, with visual fixation on a target approximately 1 m away, unpredictable and passive head turns, a peak head velocity between 150° and 250°/s, and a head turn amplitude of 20–30°. At least 10 head impulses were delivered to each plane of the semicircular canal. In the present study, it was considered abnormal if the horizontal vHIT gain was <0.8, the vertical vHIT gain was <0.7, and corrective saccades appeared (10).

### 2.4. Dizziness handicap inventory (DHI)

The DHI is standard questionnaire comprising 25 questions that quantitatively evaluates the degree of handicap in the daily life of patients with vestibular disorders (11). Patients are asked to rate the frequency by which their handicap affects them with one of three responses: no (0 points), sometimes (2 points), or always (4 points). The total scores (T-DHI) range from 0 to 100, with higher scores indicating greater disability. DHI scores can be further subdivided into physical (P-DHI, 28 points), functional (F-DHI, 36 points), and emotional (E-DHI, 36 points) subscores.

### 2.5. Hospital anxiety and depression scale (HADS)

The Hospital Anxiety and Depression Scale (HADS) is a self-report tool comprising 14 questions and is widely used to screen for anxiety and depression in medical outpatient settings (12). Each item is rated from 0 to 3 points, yielding summed scores of 0 to 42; higher scores are related to an increased likelihood that the patient could be classified as anxious or depressed. The HADS consists of two subscales: the anxiety subscale of HADS (HADS-A, 21 points) and the depression subscale of HADS (HADS-D, 21 points).

### 2.6. Niigata PPPD Questionnaire (NPQ)

The Niigata PPPD Questionnaire (NPQ) evaluates the degree of symptom exacerbation in PPPD using three factors: upright posture/walking, movement, and visual stimulation (13, 14). Each factor was assessed using four questions, for a total of 12 questions. Each question was rated on a scale of 0 (no symptoms) to 6 (intolerable), so the maximum score for each factor was 24, and the maximum score for the three factors combined was 72.

### 2.7. Stabilometry

We used the UM-BARIII (UNIMEC Co. Ltd., Japan) for stabilometric recordings. Stabilometric measurements were performed in both eyes-open and eyes-closed conditions for 60 s each. The outcome measures were the total length of the path, center of pressure (COP) area of sway movement, and Romberg ratio. The Romberg ratio was calculated by dividing the COP area of the eyes-closed condition by that of the eyes-open condition. The measured data were recorded at a 100-Hz sampling rate.

### 2.8. Statistical analyses

Descriptive data were expressed as mean ± standard deviation (SD), number (n), or percentage (%). Comparisons between the two groups were performed using Student's independent samples *t*-test, Mann–Whitney *U*-test for non-normal distribution, and Pearson's chi-square test for categorical variables. For comparisons among three groups, a one-way ANOVA was performed in the case of a normal distribution, followed by Tukey's test, and a Kruskal–Wallis test followed by Bonferroni's test in the case of a non-normal distribution. Statistical analyses were conducted using SPSS version 25 software. Statistical significance was set at a threshold of *p* < 0.05.

## 3. Results

**Figure 1:**
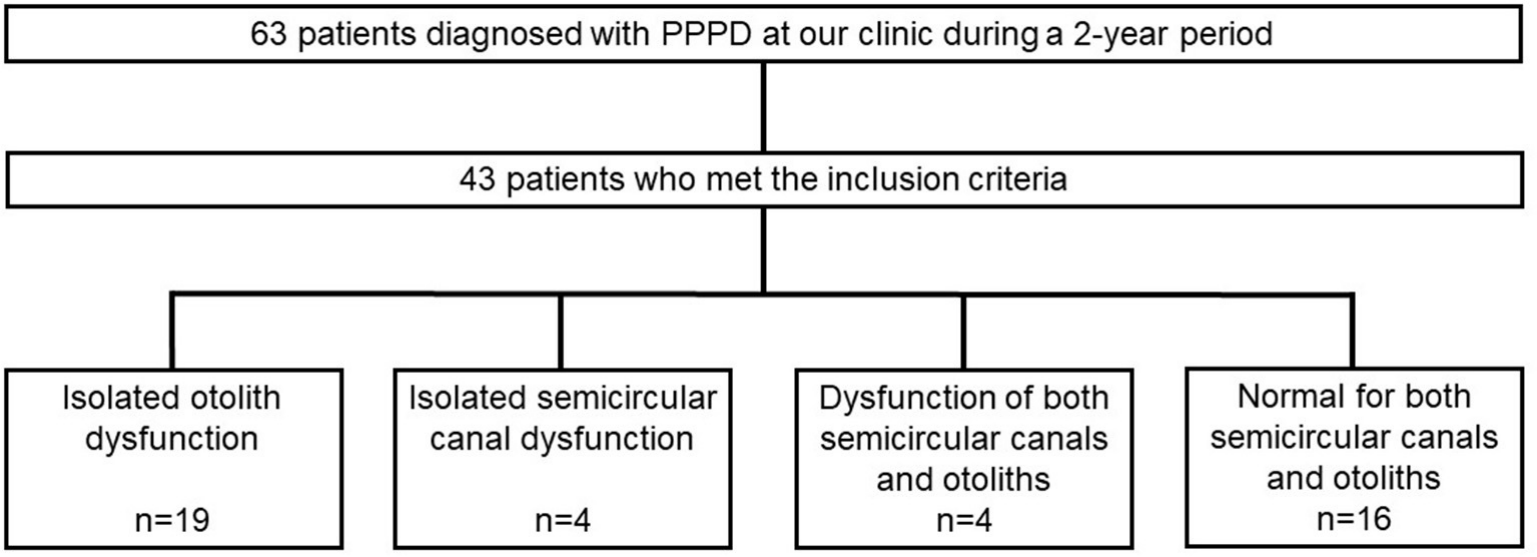
Flow diagram for study patients and classification by vestibular function tests.

The ages of the 43 patients with PPPD ranged from 23 to 78 (mean 54.0) years. Interviews with patients revealed that approximately half of them experienced acute or episodic vertigo, whereas the other half had a chronic course (Table 1). Preceding vestibular disorders as confirmed by the hospital of origin or at our clinic were present in 14 patients: 12 with BPPV (12/43, 27.9%), one with delayed endolymphatic hydrops (1/43, 2.3%), and one with Ménière's disease (1/43, 2.3%). Patients were classified into four categories based on VEMP and vHIT results: normal findings for both semicircular canals and otoliths (normal group), isolated otolith dysfunction (iOtoDys group), isolated semicircular canal dysfunction (iCanalDys group), and dysfunction of both otoliths and semicircular canals (OtoCanalDys group). Among the 43 patients with PPPD, the iOtoDys group was the largest (44.2%), followed by the normal (37.2%), iCanalDys (9.3%), and OtoCanalDys groups (9.3%; Figure 1). There were relatively more cases of preceding vestibular disorders in the iOtoDys group (8/19, 42.1%). Among them, BPPV was the most common disorder (7/19, 36.8%; Table 1). Psychiatric comorbidity diagnosed by a psychiatrist was observed in seven patients (normal group, two; iOtoDys group, four; iCanalDys, one).

**Table 1:** Diagnosis of preceding vestibular disorders and vertigo attacks in patients with PPPD.

	**iOtoDys (*n* = 19)**	**iCanalDys (*n* = 4)**	**OtoCanalDys (*n* = 4)**	**Normal (*n* = 16)**
Preceding vestibular disorders (*n* = 14)	8	1	1	4
BPPV (*n* = 12)	7	0	1	4
Delayed endolymphatic hydrops (*n* = 1)	0	1	0	0
Ménière's disease (*n* = 1)	1	0	0	0
Clinical course: past vertigo attacks (*n* = 21)	9	2	2	8
Single (*n* = 3)	1	0	1	1
Recurrence (*n* = 18)	8	2	1	7

BPPV, benign paroxysmal positional vertigo.

The basic attributes of the iOtoDys group were compared with those of the normal group (Table 2). There were no significant differences in age or sex between the two groups. The iOtoDys group had a significantly longer time from onset of dizziness symptoms compared to the normal group (19.0 vs. 13.5 months). There were significant differences in anxiety levels, as assessed using the HADS and the Romberg ratio values, between the two groups. The details of the VEMPs of the 19 patients in the iOtoDys groups are shown in Table 3. To extract the clinical characteristics of the iOtoDys group, we further divided the types into unilateral or bilateral and damage of the sacculus, utriculus, or both. Seven patients (36.8%) in the iOtoDys group showed bilateral abnormalities in both VEMPs (bilateral type), and 12 patients (63.2%) had unilateral abnormalities (unilateral type). There were no significant differences in mean DHI, HADS, or NPQ scores among the bilateral, unilateral, and normal groups (Table 4). The normal group showed a significant increase in the Romberg ratio compared to the other two groups. Anxiety scores assessed using the HADS tended to be higher in the normal group than in the other two groups, but the difference was not significant in the three-group comparison.

**Table 2:** Basic patient attributes.

	**iOtoDys group (*n* = 19)**	**Normal group (*n* = 16)**	**P-value**
Age, mean (SD) (years)	52.7 (15.4)	52.3 (13.2)	0.922^a^
Sex (male/female)	3/16	5/11	0.248^b^
Duration from onset median (min-max) (month)	19.0(5-133)	13.5(5-77)	0.031^c^
T-DHI (mean ± SD)	48.5 ± 22.9	48.0 ± 14.3	0.961^c^
HADS anxiety (mean ± SD)	6.4 ± 3.2	8.6 ± 3.0	0.044^c^
HADS depression (mean ± SD)	6.3 ± 3.9	6.6 ± 2.7	0.635^c^
NPQ	30.1 ± 12.9	35.0 ± 16.0	0.193^c^
Romberg ratio	1.3 ± 0.7	2.0 ± 0.5	< 0.001^a^

^a^*t*-test; ^b^ χ^*S*^2-test; ^c^Mann–Whitney *U*-test. T-DHI, total Dizziness Handicap Inventory; HADS, Hospital Anxiety and Depression Scale; NPQ, Niigata PPPD Questionnaire.

**Table 3:** Clinical characteristics of patients with isolated otolith dysfunction.

**ID**	**Age**	**Sex**	**Duration (month)**	**cVEMP**		**oVEMP**		**DHI**	**NPQ**	**Preceding disorders**	**Past vertigo attacks**	**Psychiatric comorbidity**
				**Right**	**Left**	**Right**	**Left**					
1	35	F	100	–	–	–	–	82	43	Cerebellar infarction		
2	78	F	6	+	+	–	–	78	38	No specific precipitants		
3	49	M	40	Decreased	+	+	+	60	40			Panic disorder
4	58	F	6	–	+	+	+	78	36	BPPV	Single	
5	42	F	9	Decreased	+	+	+	24	26	BPPV	Recurrence	
6	29	F	7	–	+	+	+	32	20			Panic disorder
7	53	F	5	Decreased	+	Decreased	+	62	36		Recurrence	
8	78	F	19	+	+	–	–	22	33	No specific precipitants		
9	38	F	17	Decreased	+	Decreased	+	76	45	Ménière's disease	Recurrence	
10	61	F	17	decreased	+	+	+	14	26	No specific precipitants		
11	37	F	47	+	+	–	+	30	26			Dysautonomia
12	57	M	22	+	+	–	–	26	12			Depression
13	58	F	80	–	+	–	–	26	42	BPPV	Recurrence	
14	44	F	45	+	+	–	+	46	38	BPPV^(*1)^	Recurrence	
15	66	F	133	Decreased	+	+	+	36	44	No specific precipitants		
16	40	M	83	+	–	–	–	38	21	BPPV	Recurrence	
17	70	F	69	–	+	–	+	76	9	BPPV^(*1)^	Recurrence	
18	73	F	9	–	+	–	–	68	36	BPPV	Recurrence	
19	36	F	18	Decreased	+	Decreased	+	48	32	No specific precipitants		

Duration, Duration from the onset of unsteadiness or non-spinning vertigo; cVEMP, cervical VEMP; oVEMP, ocular VEMP; F, female; M, male, +, reaction; –, absent; decreased for cVEMP, ARR > 34.0%, decreased for oVEMP, ARR>31.6%; DHI, Dizziness Handicap Inventory; NPQ, Niigata PPPD Questionnaire; BPPV, benign paroxysmal positional vertigo. ^(*1)^Diagnosed at our clinic.

**Table 4:** Three-group comparison of the bilateral iOtoDys, unilateral iOtoDys, and normal groups.

	**Bilateral iOtoDys mean (SD) (*n* = 7)**	**Unilateral iOtoDys mean (SD) (*n* = 12)**	**Normal mean (SD) (*n* = 16)**	**P-value**
**Diagnosis and number of preceding vestibular disorders in patients with PPPD**				
Total (*n* = 12)	3	5	4	
BPPV (*n* = 11)	3	4	4	
Ménière's disease (*n* = 1)	0	1	0	
**DHI**				
T-DHI	56.0 (26.4)	44.2 (20.6)	48.0 (19.1)	0.487
P-DHI	17.7 (8.8)	13.7 (6.1)	14.3 (6.6)	0.557
F-DHI	19.7 (10.8)	13.5 (9.2)	15.8 (7.7)	0.303
E-DHI	18.6 (8.3)	17.0 (9.2)	18.0 (8.0)	0.791
**HADS**				
Anxiety	7.6 (2.7)	5.7 (3.3)	8.6 (3.0)	0.064
Depression	8.7 (3.5)	4.9 (3.6)	6.6 (2.7)	0.053
**NPQ**				
Total	31.3 (10.9)	32.0 (10.1)	35.0 (16.0)	0.773
Upright posture/walking	8.6 (5.3)	9.6 (4.9)	11.6 (5.9)	0.408
Movement	11.9 (2.7)	11.3 (4.3)	11.3 (5.6)	0.959
Visual stimulation	10.9 (4.2)	11.2 (4.4)	12.1 (7.5)	0.880
**Stabilometry**				
**Eye open**				
COP area (cm^2^)	6.3 (2.9)	4.9 (2.7)	5.9 (4.7)	0.724
Total locus length (cm)	98.9 (35.9)	75.8 (13.6)	101.6 (44.9)	0.081
**Eye close**				
COP area (cm^2^)	10.0 (10.3)	6.1 (4.7)	11.4 (8.2)	0.229
Total locus length (cm)	129.2 (76.7)	103.8 (25.4)	170.6 (88.5)	0.05
Romberg ratio	1.3 (0.8)^(*1)^	1.2 (0.6)^(*2)^	2.0 (0.5)^(*1)^^(*2)^	0.004

BPPV, benign paroxysmal positional vertigo; DHI, Dizziness Handicap Inventory; T-DHI, total DHI; P-DHI, physical DHI; F-DHI, functional DHI; E-DHI, emotional DHI; HADS, Hospital Anxiety and Depression Scale; NPQ, Niigata PPPD Questionnaire. ^(*1)^ Significant difference between bilateral iOtoDys and normal groups. ^(*2)^ Significant difference between unilateral iOtoDys and normal groups.

Eight patients (42.1%) in the iOtoDys group showed abnormalities in both cVEMP and oVEMP, either unilaterally or bilaterally (both sacculus and utriculus damage type), and 11 patients (57.9%) had abnormalities in either cVEMP or oVEMP either unilaterally or bilaterally (either sacculus or utriculus damage type; Table 5). In a three-group comparison among the both sacculus and utriculus damage group, the either sacculus or utriculus damage group, and the normal group, the mean T-DHI, F-DHI, and E-DHI scores were significantly higher for the both sacculus and utriculus damage type than for the either sacculus or utriculus damage type. Anxiety scores on the HADS were significantly higher in the normal group than in the iOtoDys group with either sacculus or utriculus damage. The normal group showed a significant increase in the Romberg ratio compared to the two other groups. Upright posture/walking and visual stimulation as symptom-exacerbating factors assessed by the NPQ tended to be higher score in the normal group than in the other two groups, but this difference was not significant in the three-group comparison.

**Table 5:** Three-group comparison of the both sacculus and utriculus damage iOtoDys group, the either sacculus or utriculus damage iOtoDys group, and the normal group.

	**Both sacculus and utriculus damage mean (SD) (*n* = 8)**	**Either sacculus or utriculus damage mean (SD) (*n* = 11)**	**Normal mean (SD) (*n* = 16)**	**P-value**
**Diagnosis and number of preceding vestibular disorders in patients with PPPD (n)**				
Total (*n* = 12)	5	3	4	
BPPV (*n* = 11)	4	3	4	
Ménière's disease (*n* = 1)	1	0	0	
**DHI**				
T-DHI	66.0 (15.7)^(*1)^	35.8 (18.8)^(*1)^	48.0 (19.1)	0.008
P-DHI	18.3 (6.6)	12.9 (7.1)	14.3 (6.6)	0.256
F-DHI	23.8 (4.8)^(*1)^	10.0 (8.7)^(*1)^	15.8 (7.7)	0.005
E-DHI	24.0 (7.7)^(*1)^	12.9 (6.1)^(*1)^	18.0 (8.0)	0.013
**HADS**				
Anxiety	7.9 (2.9)	5.3 (3.0) ^(*2)^	8.6 (3.0) ^(*2)^	0.034
Depression	8.1 (2.9)	5.0 (4.1)	6.6 (2.7)	0.061
**NPQ**				
Total	32.3 (11.8)	31.4 (10.1)	35.0 (16.0)	0.771
Upright posture/walking	9.5 (5.9)	9.0 (4.4)	11.6 (5.9)	0.432
Movement	11.5 (4.8)	11.5 (2.9)	11.3 (5.6)	0.995
Visual stimulation	11.3 (3.2)	10.9 (5.0)	12.1 (7.5)	0.879
**Stabilometry**				
**Eyes open**				
COP area (cm^2^)	5.6 (2.6)	5.4 (3.0)	5.9 (4.7)	0.945
Total locus length (cm)	80.1 (14.6)	87.7 (32.2)	101.6 (44.9)	0.394
**Eyes closed**				
COP area (cm^2^)	7.5 (5.7)	7.7 (8.6)	11.4 (8.2)	0.391
Total locus length (cm)	100.1 (32.3)	122.3 (60.1)	170.6 (88.5)	0.075
Romberg ratio	1.3 (0.6)^(*3)^	1.2 (0.7)^(*2)^	2.0 (0.5)^(*2)^^(*3)^	0.005

BPPV, benign paroxysmal positional vertigo; DHI, Dizziness Handicap Inventory; T-DHI, total DHI; P-DHI, physical DHI; F-DHI, functional DHI; E-DHI, emotional DHI; HADS, Hospital Anxiety and Depression Scale; NPQ, Niigata PPPD Questionnaire. ^(*1)^ Significant difference between the both sacculus and utriculus damage subgroup and the either sacculus or utriculus damage subgroup. ^(*2)^ Significant difference between the either sacculus or utriculus damage subgroup and the normal group. ^(*3)^ Significant difference between the both sacculus and utriculus damage subgroup and the normal group.

## 4. Discussion

### 4.1. Key findings

In the present study, preceding vestibular disorders confirmed in the hospital of origin or at our clinic were observed in 14 of 43 patients with PPPD: BPPV was the most frequent disorder (12/43, 27.9%), which is consistent with findings of previous reports (4, 15). Approximately half of the patients with PPPD presented with otolith dysfunction. Hence, our data suggest that otolith dysfunction could be a potential factor in the development of PPPD. The majority of patients with PPPD were female in the present study (female/male ratio: 2.58/1). Previous studies have also reported female preponderance (16–18). When divided into four groups based on VEMP and vHIT findings, isolated otolith dysfunction was the most common, accounting for 44.2% of patients with PPPD. The results were similar to those reported by Murofushi et al. (4) (46%) and relatively higher than those reported by Adamec et al. (6) and Waterston et al. (7), which reported rates of 32.1 and 20.0%, respectively. There were relatively more cases of preceding vestibular disorders in the iOtoDys group. In the iOtoDys group, the characteristics differed according to the spread of lesions. Damage to both the sacculus and utriculus had a significant effect on DHI (Table 5). However, no significant difference was found in the various outcomes regarding whether otolith damage was unilateral or bilateral (Table 4). On the other hand, damage to both the sacculus and utriculus had relatively lower effects on symptom exacerbation during upright posture/walking and movement according to NPQ results. These facts might suggest that isolated otolith dysfunction is not the only major precipitant of PPPD, but that compensatory insufficiency due to the coexisting deficits exacerbates dizziness symptoms in PPPD. The prevalence of otolith dysfunction and resulting dizziness symptoms in the general population should be investigated to further analyze the impact of otolith dysfunction on PPPD. In the present study, we evaluated the function of the semicircular canals using the vHIT. The vHIT has the advantage of evaluating the function of each of the three semicircular canals. However, although the vHIT is known to have higher diagnostic specificity than the caloric test, it has lower sensitivity (19). Incorporating the caloric test into the evaluation may allow for the identification of distinct clinical features of the iOtoDys group of patients with PPPD.

In this study, 16 of 43 PPPD patients (37.2%) had normal VEMP and vHIT results. HADS scores tended to be higher in the normal group compared to the iOtoDys group in our study. In addition, DHI scores were also significantly higher in the normal group compared to the either sacculus or utriculus damage group. Contrary to expectations, no patient with both sacculus and utriculus damage type in the iOtoDys group had psychiatric comorbidities (Table 3). Habs et al. (15) reported that 55% of patients with PPPD are idiopathic without preceding vestibular disorders but showed psychiatric comorbidities such as anxiety and depressive disorders, so the psychiatric comorbidities can greatly affect the pathogenesis of PPPD. In general, the Romberg ratio is considered to be higher in the presence of vestibular dysfunction; however, the Romberg ratio was significantly higher in the normal group compared to the iOtoDys group in our study. Dizzy patients with anxiety tend to have greater postural sway in an erroneous or conflicting visual environment (14, 20), which may be related to a favorable shift of sensory reweighting to visual cues (21). Based on these results, the Romberg ratio should be interpreted carefully in the clinical assessment since the value in normal subjects within the same age group ranges from 0.15 to 2.62 (mean ± SD, 1.39 ± 0.61) (22). Further evaluations using stabilometry using foam rubber may also be necessary.

The NPQ identifies the status of PPPD patients by dividing them into three subtypes (13). Unfortunately, we found no association between the presence of otolith damage and the particular symptom exacerbation factor of PPPD, possibly because the NPQ is more suitable for extracting a factor that is exacerbated by visual stimuli than factors that are exacerbated by an upright posture or movement (13). Further studies are required to increase the number of patients.

Pharmacotherapy, vestibular rehabilitation (VRT), and cognitive behavioral therapy (CBT) have been reported as treatment options for PPPD, and there are currently no gold standard guidelines for the treatment of PPPD (3, 23). One reason for this may be that the pathophysiology of PPPD is unclear (24). The present study suggests that vestibular testing in PPPD may lead to individualized treatment options (e.g., VRT focusing on otolith adaptive change and improving social participation for PPPD patients with otolith dysfunction).

### 4.2. Limitations

This study has several limitations. First, the sample size is small. Of the 43 patients, there were only four patients in the isolated semicircular canal dysfunction group and four patients in the both otolith and semicircular canal dysfunctions group. In the future, the sample size should be increased to allow for comparisons among the four groups. Second, the evaluation items were mainly based on questionnaires, and the performance evaluation was a stabilometry for static balance assessment. Symptoms of PPPD include chronic dizziness and non-rotational dizziness while standing or walking. Thus, the use of dynamic performance tests such as the Dynamic Gait Index (DGI), Functional Gait Assessment (FGA), and Timed Up and Go test (TUG) is recommended, which include pivoting movements and changes in direction. Third, the 10 PPPD patients in this study were older than 65 years. VEMP testing may decrease in amplitude with age in both cVEMP and oVEMP. Caution should be exercised in interpreting abnormal VEMP findings in the elderly (25, 26). Fourth, we examined oVEMP using an air-conducted stimulus but not a bone-conducted stimulus. Although oVEMP using air-conducted and bone-conducted stimuli are thought to similarly reflect the function of the utricle, which is mediated by the superior vestibular nerve, more stable responses have been reported with a bone-conducted stimulus (27, 28).

## 5. Conclusions

Our results suggest that the coexistence of both sacculus and utriculus damages may exacerbate dizziness symptoms in patients with PPPD. Patients with PPPD without vestibular dysfunction tended to have greater anxiety and sway with their eyes closed. Identification of these features using vestibular function tests may aid in the selection of effective individualized treatments.

## Data availability statement

The raw data supporting the conclusions of this article will be made available by the authors, without undue reservation.

## Ethics statement

The studies involving human participants were reviewed and approved by Medical Research Ethics Committee of Mejiro University. Written informed consent for participation was not required for this study in accordance with the national legislation and the institutional requirements.

## Author contributions

Material preparation and data collection were performed by HF, RTsu, and MA. Data analysis was performed by HF and MA. The first draft of this manuscript was written by HF and MA. TKam, HO, RTan, TKat, and RTsu critically reviewed this manuscript.
